# Reply to Wostyn, P.; Nedergaard, M. Ocular Glymphatic Dysfunction as a Potential Link Between Obstructive Sleep Apnea and Retinal Structural Changes. Comment on “Pusic Sesar et al. Multimodal Assessment of Ocular Parameters in Patients with Severe Obstructive Sleep Apnea with Emphasis on Retinal Structural Changes. *Life* 2025, *15*, 1307”

**DOI:** 10.3390/life16020215

**Published:** 2026-01-28

**Authors:** Anita Pusic Sesar, Anja Cehajic, Antonela Geber, Mia Zoric Geber, Ivan Cavar, Antonio Sesar

**Affiliations:** 1 Department of Ophthalmology, School of Medicine, University of Mostar, 88 000 Mostar, Bosnia and Herzegovina; pusicanita@gmail.com (A.P.S.); antoniosesar@yahoo.com (A.S.); 2 Department of Anesthesia and Intensive Care, University Hospital Center Mostar, 88 000 Mostar, Bosnia and Herzegovina; anjacehajic42@gmail.com; 3 Department of Ophthalmology, University Hospital Center Zagreb, 10000 Zagreb, Croatia; 4 Department of Ophthalmology, Sestre Milosrdnice University Hospital Center, 10000 Zagreb, Croatia; miazoricgeber@gmail.com; 5 Department of Physiology and Immunology, School of Medicine, University of Mostar, 88 000 Mostar, Bosnia and Herzegovina

We sincerely thank you for your thoughtful and scientifically enriching comments [1] on our recently published article, “Multimodal Assessment of Ocular Parameters in Patients with Severe Obstructive Sleep Apnea with Emphasis on Retinal Structural Changes” [2]. Your expertise in ocular glymphatic physiology provides an important framework that deepens the interpretation of our findings.

In our study, we observed significant thinning of the ganglion cell layer (GCL) in patients with severe obstructive sleep apnea (OSA) compared with healthy controls, along with a trend toward reduced retinal nerve fiber layer (RNFL) thickness that did not reach statistical significance. We noted that these alterations could be partially explained by hypoxia, vascular dysregulation, and slightly elevated intraocular pressure (IOP) [2].

Your observation that sleep plays a key role in maintaining metabolic homeostasis, with impaired sleep quality being associated with reduced glymphatic clearance of amyloid-β (Aβ) in the brain, emphasizes the importance of sleep for the removal of toxic waste products [3]. Optimal sleep is crucial for maintaining brain health, and multiple pathways, including glymphatic clearance, may be affected in sleep disorders [4]. Furthermore, the finding of retrograde glymphatic-like fluid transport from the brain toward the optic nerve introduces a compelling mechanism that may contribute to early glaucomatous damage independently of IOP. The ocular glymphatic system has been shown to facilitate the removal of amyloid-β (Aβ) and other metabolites from the retina and optic nerve, with efflux driven by the trans-lamina cribrosa pressure difference, influenced by both IOP and intracranial pressure (ICP) [5,6,7,8]. This perspective is highly relevant to our study, in which significant GCL thinning and a trend toward reduced RNFL thickness were noted in patients with severe OSA, despite the absence of significant IOP elevation.

We found your discussion regarding retrograde ocular glymphatic clearance impairment particularly insightful. As you highlighted, insufficient or fragmented sleep, common in patients with OSA, may reduce the efficiency of clearance along this pathway, leading to the accumulation of metabolic waste products at the optic nerve head and increased susceptibility to neurodegeneration, especially in normal-tension glaucoma [9]. This mechanism aligns with epidemiological data demonstrating associations between short sleep duration, insomnia, sleep apnea syndrome, and RNFL thinning. Impaired glymphatic clearance, both retrograde from the brain to the optic nerve and anterograde from the intraocular space through retinal ganglion cell axons, may contribute to early glaucomatous changes independently of IOP [10].

Additionally, your description of the recently identified anterograde ocular glymphatic clearance system provides an important complementary mechanism. As you detailed, this system relies on efflux across the lamina cribrosa driven by the trans-lamina pressure difference between IOP and ICP. Dynamic ICP fluctuations during OSA, including changes in intrathoracic pressure and venous return with subsequent venous congestion, may reduce this gradient and impede intra-axonal clearance [11]. Such pathophysiological insights offer a plausible explanation for the retinal structural alterations observed in our cohort and further support an IOP-independent association between OSA and early glaucomatous changes.

We are grateful for your comprehensive and forward-thinking contribution, and we fully agree that future studies integrating OCT-based structural assessment, polysomnography parameters, ICP/IOP dynamics, and glymphatic imaging approaches are essential to further clarify these relationships. Incorporating glymphatic biomarkers and advanced imaging modalities may help determine the contribution of ocular glymphatic dysfunction to early retinal neurodegeneration in OSA. Your perspective enriches our understanding of glaucoma pathophysiology and underscores the importance of exploring IOP-independent mechanisms in neuro-ophthalmology.
